# Besonders geschützt oder ausgestoßen? Wie Personen im Alter über 65 Jahre die Coronapandemie erlebten

**DOI:** 10.1007/s00391-022-02112-9

**Published:** 2022-09-28

**Authors:** Rosangela Aversa, Stefanie Fluri, Agnes von Wyl

**Affiliations:** Departement Angewandte Psychologie, Psychologisches Institut, Fachgruppe Klinische Psychologie und Gesundheitspsychologie, ZHAW Zürcher Hochschule für Angewandte Wissenschaften, Pfingstweidstr. 96, Postfach, 8037 Zürich, Schweiz

**Keywords:** COVID-19, Alter, Alltagsbewältigung, Mixed-Methods-Design, Altersdiskriminierung, COVID-19, Older people, Coping with everyday life, Mixed methods design, Ageism

## Abstract

**Hintergrund:**

Zu Beginn der Coronapandemie wurden auch in der Schweiz Personen im Alter ab 65 Jahren der besonders gefährdeten Bevölkerungsgruppe zugeordnet. Aufgrund vermehrter Vorerkrankungen wurde vermutet, sie seien einem erhöhten Risiko für schwere Krankheitsverläufe ausgesetzt. Dadurch rückten ältere Personen in den Fokus der Aufmerksamkeit, wodurch deren gebrechliche und hilflose Seite betont wurde. Dies lässt Fragen offen, bezüglich der Selbsteinschätzung der älteren Menschen, was ihr konkretes subjektives Befinden und Erleben während der Pandemie betrifft.

**Ziel der Arbeit:**

Die Studie ergründet die subjektive Sichtweise von Personen ab 65 Jahren und hat zum Ziel, ihre Alltagsbewältigung während der Pandemie zu untersuchen, insbesondere in Bezug auf die Auswirkungen auf ihre Lebenssituation, ihre Selbsteinschätzung als vulnerable Gruppe und ihre Ängste rund um COVID-19.

**Material und Methoden:**

Es handelt sich um eine Längsschnittstudie im Mixed-Methods-Design, bei welcher von Ende April bis Mitte Juni 2020 2‑wöchentlich ein leitfadengestütztes Telefoninterview mit geschlossenen und offenen Fragen durchgeführt wurde. Es wurden 40 Personen (m = 18, w = 22) im Alter zwischen 65 und 90 Jahren zu verschiedenen Aspekten der Alltagsbewältigung während der Coronapandemie und ihren Folgen befragt. Die quantitativen Daten wurden deskriptiv ausgewertet. Die qualitativen Daten wurden mittels strukturierter Inhaltsanalyse mit induktiver Kategorienbildung evaluiert.

**Ergebnisse:**

Die Studie zeigte, dass sich das Alltagsleben der Befragten trotz des Lockdowns zu Hause kaum veränderte. Auch wurde mehrheitlich über eine gute Stimmungslage berichtet. Als große Belastung wurden die sozialen Einschränkungen erlebt. Die Einordnung als Risikogruppe wurde als undifferenziert und willkürlich empfunden. Angst oder Sorgen hinsichtlich einer Ansteckung mit dem Coronavirus waren wenig vorhanden.

**Diskussion:**

Die Befragten schienen die Krise deutlich besser bewältigt zu haben, als der öffentliche Diskurs nahelegte. Aktivitäten und Routinen können als Strategien im Alltag schützend gewirkt haben. Eine homogene Einteilung der älteren Personen als Risikogruppe vernachlässigt deren Ressourcen und fördert sowohl negative Stereotype als auch Altersdiskriminierung.

**Zusatzmaterial online:**

Zusätzliche Informationen sind in der Online-Version dieses Artikels (10.1007/s00391-022-02112-9) enthalten.

## Abstract (en)

**Background:**

In the COVID-19 pandemic, in Switzerland people aged 65+ years were categorized as a high-risk group, as they are at higher risk of severe COVID-19 due to higher rates of underlying health conditions. This focused attention on older adults, mostly with respect to frailty and helplessness. What is lacking is older adults’ subjective views, including their state of mind and their experiences.

**Objective:**

This study explores the subjective view of older adults and aims to examine their everyday coping during the pandemic, particularly the concrete impacts on their lives, their self-assessment as a vulnerable group, and fears about COVID-19.

**Material and methods:**

The longitudinal study was conducted from April to June 2020 using a mixed methods design comprising both closed and open-ended questions. We conducted guided biweekly interviews by telephone with 40 people (22 were women) aged 65–90 years. The quantitative data included descriptive analyses. The qualitative data were evaluated by structured content analysis with inductive category formation.

**Results:**

The respondents reported little change in their everyday lives. Overall, a good mood was reported. Social restrictions were experienced as a great burden. The respondents perceived their classification as a risk group as undifferentiated and arbitrary. Fear or worry about contracting the coronavirus was low.

**Discussion:**

Respondents appear to have coped much better with the crisis than public discourse suggested. Activities and routines as well as support services may have been protective as strategies in everyday life. The homogeneous classification of older adults as an at-risk group ignores their resources and promotes both negative stereotypes and ageism.

## Einleitung

**Figure 1:**
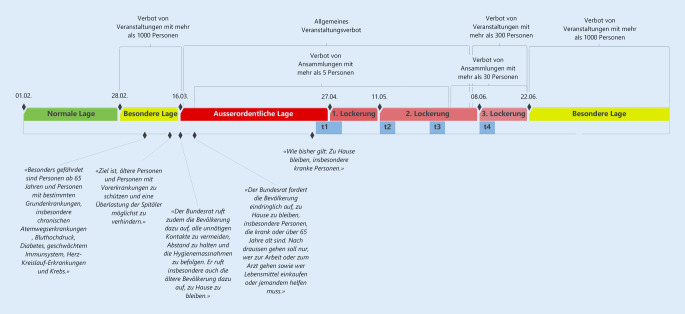

Im Frühjahr 2020 nahm die Coronapandemie die Schweiz in Beschlag. Am 13.03.2020 wurde die Kampagne „Bleiben Sie zu Hause“ lanciert, und alle, v. a. Personen ab 65 Jahre und solche mit Vorerkrankungen, wurden dazu angehalten, zu Hause zu bleiben. Personen ab 65 Jahre wurden der besonders gefährdeten Gruppe, der sog. Risikogruppe, zugeordnet: Sie seien aufgrund vermehrter Vorerkrankungen einem zusätzlichen Risiko ausgesetzt, sich mit dem Coronavirus anzustecken und somit eher von schweren Krankheitsverläufen betroffen [3]. Dadurch rückten besonders zu Beginn der Pandemie ältere Personen in den Fokus der Aufmerksamkeit, und es wurden altersspezifische Schutzmaßnahmen ausgesprochen: Die ältere Bevölkerung wurde dazu angehalten, mit niemandem außerhalb desselben Haushalts Kontakt zu haben, keine öffentlichen Verkehrsmittel zu benutzen und das Einkaufen zu vermeiden. Diese restriktiven Maßnahmen wurden 6 Wochen später gelockert (Abb. 1).

Obwohl das Medianalter der an oder mit COVID-19 verstorbenen Personen in der Schweiz relativ konstant bei 85 Jahren lag, wurden alle Personen über 65 Jahre der Risikogruppe zugeteilt [3]. Verschiedentlich wurde kritisiert, dass diese pauschale Zuteilung einer multidimensionalen Definition von Alter sowie den heterogenen Lebensläufen nicht gerecht wird [24] und sich allein auf das gesellschaftlich institutionalisierte Rentenalter beschränkt. Eine solche Grenzziehung kann Altersdiskriminierung und Altersstereotypen begünstigen [20] und die betroffene Gruppe isolieren.

Die Frage stellte sich, wie ältere Personen die Coronapandemie erlebten und wie sie deren Auswirkungen in ihrem Alltag bewältigten. Aus der bisherigen Forschung weiß man, dass ältere Erwachsene im Alltag von weniger negativen Emotionen berichten [7], stressreiche Erlebnisse als weniger unangenehm empfinden und tiefere Werte bei Angst und Depression erzielen [14]. Aber gilt dies auch in einer Krisenzeit, in der zudem die Gesundheit v. a. älterer Personen im Fokus ist? Verschiedene Forschungsteams gingen der Frage nach, ob sich ältere Personen während der Coronapandemie bezüglich verschiedener Faktoren von jüngeren Personen unterscheiden. So zeigte eine Untersuchung, dass ältere Erwachsene zwar eher damit rechnen, an COVID-19 zu sterben, sich aber weniger Sorgen machen, daran zu erkranken, in Quarantäne gehen zu müssen oder kein Geld mehr zu haben; auch zeigten sie geringere Depressions- und Angstwerte [8]. Außerdem bewerteten sie die eigene Bewältigung der Situation positiver als jüngere Personen [9]; wichtig für die Bewältigung sei, beschäftigt zu sein, soziale Unterstützung zu bekommen und eine positive Einstellung zu haben. Allerdings waren diese beiden Studien Querschnitterhebungen. Auch wurden keine spezifischen Veränderungen im Alltag aufgrund von COVID-19 oder mögliche Anpassungen im Laufe der Pandemie mit sich verändernden Einschränkungen untersucht.

In der Schweiz informierte ab Beginn der Coronapandemie ein kontinuierliches Monitoring zu verschiedenen Aspekten im Zusammenhang zur Coronapandemie [5]. Die repräsentative Befragung zu gesundheitlichen, sozialen, politischen und wirtschaftlichen Implikationen fand zum ersten Mal im März 2020 statt. Daran anknüpfend hatte die vorliegende Untersuchung das Ziel, diese Ergebnisse bei älteren Personen zu vertiefen: Wie erlebten die Betroffenen die Pandemie? Welche Ängste spielten eine Rolle? Wie bewältigten sie ihren Alltag, und was waren die konkreten Auswirkungen? Dabei interessierte die Sicht der Betroffenen und ihr subjektives Erleben über den Zeitraum vom Lockdown bis zur (vorübergehenden) zunehmenden Aufhebung der Verhaltensempfehlungen.

## Methoden

Um einen vertieften Einblick zum Erleben und Verhalten von Personen 65+ während der ersten Monate der Coronapandemie zu erhalten, wurde eine Studie im Mixed-Methods-Design mit 4 Erhebungszeitpunkten durchgeführt. Das Längsschnittdesign erlaubte, die Auswirkungen der – v. a. während der ersten Pandemiezeit – sehr einschränkenden Maßnahmen für die ältere Bevölkerung abzubilden. Zu Beginn der Befragung war nicht absehbar, wie die pandemische Situation zum jeweiligen Zeitpunkt der Interviews sein wird. Die Hauptthemen *Auswirkungen auf die eigene Lebenssituation, Zugehörigkeit zur Risikogruppe* sowie *Angst vor COVID und andere Sorgen* wurden in Telefoninterviews sowohl mit geschlossenen als auch mit mehreren offenen Fragen erhoben. Die Tab. 1, 2 und 3 geben einen Überblick über die Hauptthemen und die zugeordneten Fragen (quantitativ und qualitativ). Es handelt sich um Themen, die zu Beginn der Pandemie breit diskutiert wurden und als verstärkende Faktoren für psychische Beeinträchtigungen erachtet wurden. Die Erhebung dauerte von Ende April 2020 (KW18, t0) bis Mitte Juni 2020 (KW24, t5); die Interviews erfolgten ungefähr alle 2 Wochen.

**Table 1:** 

Auswirkungen auf die eigene Lebenssituation		
	Quantitative Fragen	Qualitative Fragen
Stimmungslage	Erzählen Sie mal, wie es Ihnen zurzeit geht. (Fünfstufige Likert-Skala: sehr schlecht bis sehr gut)	Induktiv aus weiteren Erzählungen
Alltagsgestaltung und -bewältigung	Wofür haben Sie diese Woche Ihre Wohnung/Ihr Haus verlassen? (Diverse Antwortmöglichkeiten, s. Zusatzmaterial online)	Erzählen Sie mir von Ihrem Alltag in ihrer Wohnung/im Altersheim. Ich möchte mir gerne vorstellen, wie ein üblicher Tag jetzt seit den Einschränkungen wegen der Coronakrise bei Ihnen aussieht
		Was ist der Unterschied zum Alltag vor der Coronakrise?
		Was fehlt Ihnen im Alltag? Was ist am schwierigsten zu ertragen?
		Was fällt Ihnen einfach, was ist schwierig?
Kontakt zu anderen Personen	Mit wie vielen Menschen, die nicht mit Ihnen zusammenleben, hatten Sie diese Woche näheren Kontakt (länger als 15 min, näher als 2 m)? Anzahl der Begegnungen: 0; 1–2; 3–5; 6–10; 11–20; 21+	–
Unterstützung	–	Von wem und wofür haben Sie Hilfe oder Unterstützung erhalten?
		Was fehlt Ihnen in Bezug auf Unterstützung?
		Welche Unterstützung würden Sie sich wünschen?

**Table 2:** 

Zugehörigkeit zur Risikogruppe
Qualitative Fragen
Wie erleben Sie die Zuweisung in die Hochrisikogruppe? Denken Sie, dass Sie nicht dazu gehören?
Es interessiert mich, wie Sie sich durch die besonderen Bestimmungen fühlen
Welche Reaktionen der Bevölkerung erleben Sie?
Haben Sie in dieser Zeit Beschuldigungen oder Vorurteil gegenüber älteren Personen wahrgenommen?
Wie reagieren Sie auf diese Reaktionen?

**Table 3:** 

Angst vor COVID-19 und zu anderen Sorgen	
Quantitative Fragen	Qualitative Fragen
Wie schätzen Sie die Gefährlichkeit des Coronavirus (COVID-19) für sich selber ein? (Vierstufige Likert-Skala: Ich mache mir keine Gedanken darüber. Ich gehe von einem milden Verlauf aus. Ich befürchte einen schweren Verlauf. Ich befürchte einen tödlichen Ausgang)	Haben Sie persönlich Angst?
	Was macht Ihnen besonders Angst? Wie gehen Sie mit Ihrer Angst um?

Die Stichprobe setzte sich aus 40 Personen aus der deutschsprachigen Schweiz zusammen. Wichtigstes Einschlusskriterium war das Alter, welches über 65 Jahren liegen musste. Tab. 4 zeigt die Verteilung der Rekrutierungsmerkmale.

**Table 4:** 

	*n*	%
*Geschlecht*		
Männer	18	45
Frauen	22	55
*Alter*		
65 bis 75 Jahre	22	55
76 bis 90 Jahre	18	45
*Wohnsituation*		
Alleine	16	40
Mit Partner	24	60
*Region*		
Stadt	26	65
Land	14	35

*n* = 40

Die Rekrutierung erfolgte über diverse Organisationen (u. a. Caritas, Pro Senectute). Die freiwillige Teilnahme wurde mit einer Einverständniserklärung bestätigt.

Sämtliche Interviews wurden digital aufgezeichnet und mithilfe der Software MAXQDA [15] transkribiert. MAXQDA ist eine Software, die sowohl den Transkriptions – wie auch den Analyseprozess unterstützt. Die Aufbereitung und Auswertung der transkribierten Interviews erfolgte mittels strukturierender Inhaltsanalyse mit induktiver Kategorienbildung [16]. Um die zentralen Inhalte herauszufiltern, wurde das Textmaterial in mehreren Arbeitsschritten systematisch reduziert. Zunächst wurden fallweise alle inhaltstragenden Äußerungen der Befragten markiert. MAXQDA erlaubt, die markierten Inhalte zu exportieren; in der Folge wurden mit diesen weitergearbeitet. Fallübergreifend wurden Textstellen mit vergleichbarem Inhalt zu Kategorien zusammengeführt. Parallel zu dieser individuell durchgeführten Inhaltsanalyse fanden regelmäßig Forschungswerkstätten der beteiligten Forscherinnen statt [17]. Die Auswertung und Ergebnisse wurden im Sinne einer kommunikativen Validierung kritisch überprüft und diskutiert. Das erarbeitete Kategoriensystem wurde von der ersten und der zweiten Autorin überprüft und die Transkripte unabhängig voneinander kodiert. Die Forschungsleiterin rücküberprüfte diese. Die einzelnen Abschnitte im Ergebnisteil repräsentieren die Hauptkategorien. Die quantitativen Daten wurden mit deskriptiver Statistik ausgewertet.

## Ergebnisse

### Auswirkungen auf die eigene Lebenssituation

**Figure 2:**
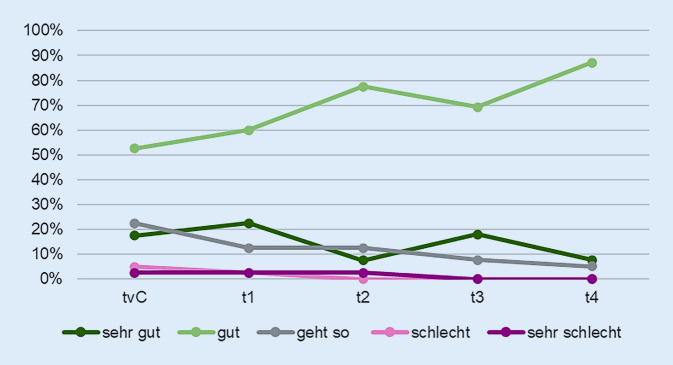

Die Auswertung der geschlossenen Fragen ergab, dass die Coronapandemie für viele der befragten Personen keine negative Auswirkung auf die Stimmungslage mit sich brachte. Abb. 2 zeigt die Mittelwerte der Veränderung der Stimmungslage.

Die Einschränkungen des öffentlichen Lebens erlebten nur wenige der Befragten als gravierend. Die Gestaltung des Alltags mit Spaziergängen, Lektüren, Haushaltserledigungen und Kochen als Hauptaktivitäten gehörte für viele aufgrund der Pensionierung bereits vor der Pandemie zum Alltag. Selbst Ende März 2020 (t1) verließen 75 % aller Befragten das Haus zum Spazierengehen und 45 % für Lebensmitteleinkäufe. Das Treffen von Freunden (30 %) sowie Arztbesuche (30 %) und Ausflüge (23 %) wurde aber deutlich reduziert. Eine Minderheit (15 %) verließ das Haus nicht. Anfang Juni 2020 (t4) waren Spaziergänge weiterhin eine häufige Alltagsaktivität (75 %). Einkaufen (90 %), Treffen von Freunden (28 %) und Ausflüge an andere Orte (54 %) nahmen zu. Nur 3 % der befragten Personen verließen das Haus nach wie vor nicht. Die Mehrheit der Befragten verließ das Haus über die 4 Befragungszeitpunkte immer öfter und beteiligte sich vermehrt an Aktivitäten.

**Figure 3:**
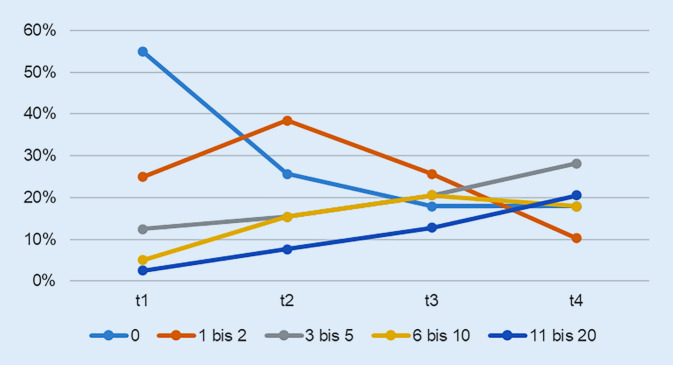

Die Befragten erklärten, weshalb sie ihre Tagesstruktur und Routinen mehrheitlich beibehalten konnten: „Ja, wissen Sie, ich bin ja pensioniert. Da hat sich nicht wahnsinnig viel geändert, nicht wahr“ (TN37). Sie beschrieben aber auch, wie sehr die sozialen Kontaktbeschränkungen mit einer deutlichen Belastung verbunden waren. Das Zusammensein mit der Familie und Freunden wurde stark vermisst. Der Austausch via soziale Medien war als Kompensation nicht zufriedenstellend und teilweise ermüdend: „Das Schwierigste sind einfach die fehlenden Kontakte, das Menschliche, das einem Nahekommen. Das Telefonieren ist ein Ersatz, aber es ist nicht das, was ich gerne hätte“ (TN11). Der fehlende physische Kontakt zu den Enkelkindern und die fehlende Teilhabe an deren Entwicklung wurde als schmerzlich erlebt: „Ich würde gerne meine Enkel wieder einmal drücken und sie näher haben. Man schreibt sich und sieht sich ab und zu auf Distanz. Aber ich will schon, dass sich das wieder normalisiert“ (TN09). Das Wegfallen von öffentlichen Anlässen und Besuchen von Restaurants, Vereinen oder bestimmten Hobbys wurden als Limitierung erlebt. Abb. 3 zeigt die Anzahl der physisch näheren Kontakte (länger als 15 min, näher als 1,5 m) außerhalb des engen Umfelds in den vergangenen 7 Tagen.

Die große Mehrheit der befragten Personen berichtete über Unterstützungsleistungen in Form von Einkäufen von Nachbar*innen oder Familienmitgliedern. Die solidarischen Initiativen wurden geschätzt und mit Emotionen wie Freude, Erleichterung sowie auch Erstaunen begleitet: „Ja, also ich muss sagen, es ist wirklich schön, von wie vielen Personen ich Unterstützung bekam. Weißt du, auch von solchen, die ich überhaupt nicht kenne“ (TN4). Durch die erhaltene Unterstützung fühlten sich einige nicht allein gelassen: „Man erhält so viel Hilfe, ich fühle mich überhaupt nicht allein“ (TN04). Einige berichteten von schlechtem Gewissen oder persönlichen Schwierigkeiten, wie z. B. dem Rollenwechsel hin zur hilfesuchenden Person oder dem Angewiesensein auf Hilfsangebote. Einzelne berichteten von Familienmitgliedern, welche ihnen den Ausgang verboten, sodass sie gezwungen waren, praktische Hilfen, wie z. B. das Einkaufen, anzunehmen. Einige verzichteten freiwillig auf diese Angebote und betonten ihre Selbstständigkeit: „Es hat mir jetzt jemand aus der Apotheke angerufen, ob ich Hilfe benötige […] Aber wir brauchen das nicht. Ich bin selbstständig“ (TN35).

### Zugehörigkeit zur Risikogruppe

Die im Zusammenhang mit der Coronapandemie definierte Vulnerabilität von Personen ab 65 Jahre und deren Einordnung als Risikogruppe wurde von den befragten Personen als undifferenziert empfunden. Zwei relevante Faktoren wurden diskutiert: der Gesundheitszustand und das Alter. Diejenigen, die von coronagefährdeten Vorerkrankungen betroffen waren, gaben an, zur Risikogruppe zu gehören. Das Alter von 65 Jahren als Begründung zur Einteilung wurde als unberechtigt und willkürlich wahrgenommen: „Also mit 65, das ist eine willkürliche Wahl, eine Bestimmung. Ich bin 66 und denke, es kann für einige Personen gefährlicher sein als für mich, auch solche die jünger sind und einige Voraussetzungen erfüllen“ (TN01). Vor allem die knapp über 65-Jährigen waren nicht der Meinung, zur Risikogruppe zu gehören.

Diese Einordnung hatte Einschränkungen und Konsequenzen im Zusammenleben, welche unterschiedlich wahrgenommen wurden. Einige machten den Bezug zur Gesundheit und Selbstständigkeit: „Ich fühle mich ein bisschen diskriminiert, weil ich jetzt zu dieser Risikogruppe gehöre, obwohl ich eigentlich verhältnismäßig gesund bin. Und auch noch selbstständig“ (TN23). Wenige berichteten über Diskriminierung und Ausgrenzung: „Ja es ist teilweise für mich diskriminierend, dass man auf den Alten herumhackt“ (TN12). Andere differenzierten zwischen Schutz und Diskriminierung, wobei in der Regel Ersterer überwog. Eine Person berichtete über eine direkte diskriminierende Erfahrung: „Ich wurde auch schon angepöbelt von Jungen, die schon ein wenig angetrunken und übermütig waren. Die haben mich von Weitem angepöbelt: ‚Alte, geh nach Hause, du hast hier nichts zu suchen‘“ (TN33). Ansonsten wurden altersdiskriminierende Aussagen nur von Dritten und in den Massenmedien gehört. Der in den Medien diskutierte Kampf „Alte gegen Junge“ wurde ab und zu erwähnt.

### Angst vor COVID-19 und andere Sorgen

Zu t1 befürchteten einige der befragten Personen bei einer Ansteckung mit COVID-19 einen tödlichen Ausgang (13 %) oder schweren Verlauf (15 %). Ein Großteil (44 %) machte sich hingegen keine Gedanken darüber. Andere gingen von einem milden Verlauf (28 %) aus. Zu t4 befürchtete niemand mehr einen tödlichen Verlauf, hingegen nahmen die Befürchtungen über einen schweren Verlauf zu (34 %).

Zur Angst vor einer Ansteckung mit dem Coronavirus kamen zögerliche Antworten. Das Wort „Angst“ wurde sehr oft mit „Respekt“ korrigiert: „Diese Coronakrise, das ist wirklich eine sehr gefährliche Sache. Ich habe gewissen Respekt, also keine Angst, aber Respekt davor“ (TN40). Die Leute gaben an, kaum Angst zu haben, sich mit dem Virus zu infizieren. Größer als die Sorge vor Ansteckung war einen möglichen schlimmen Verlauf auf der Intensivstation und die Art und Weise, wie die restliche Lebenszeit gelebt werden müsste. Als Grund für fehlende Sorge berichteten einige über die vorhandene Patientenverfügung, das Einhalten der Schutzmaßnahmen und ihren guten Gesundheitszustand: „Hmm, nein [keine Angst], denn ich habe eine Patientenverfügung und ich weiß, dass ich dann nicht so große Eingriffe haben will“ (TN18). Einige zeigten eine lockere bzw. optimistische Haltung bezüglich des Ansteckungsrisikos: „Wenn es mich dann doch erwischt, dann muss ich mich mit dieser Situation halt abfinden. Aber darüber mache ich mir jetzt noch keine Sorgen“ (TN11). Das fortgeschrittene Alter und die bereits erfolgte Auseinandersetzung mit dem Tode äußerte sich in einer Gelassenheit und positiven Haltung gegenüber dem Weiterleben.

In einzelnen Interviews wurden weitere Bedenken wie psychische und physische Beeinträchtigungen, Verlustängste sowie allgemeine politische und wirtschaftliche Konsequenzen geäußert. Die „Liquidierung der Alten“ und der Verlust der Anerkennung älterer Personen wurden ebenfalls erwähnt.

## Diskussion

Im Rahmen der nationalen Schutzmaßnahmen gegen COVID-19 wurden zu Beginn alle älteren Personen ab 65 Jahre als besonders gefährdete Gruppe deklariert [4]. Die Schutzmaßnahmen empfahlen dringend, direkte soziale Kontakte und Außerhausaktivitäten zu vermeiden. Unsere Studie beleuchtete das Erleben sowie die Alltagsbewältigung von Personen 65+ über den Zeitraum von Ende April bis Mitte Juni 2021. Die Bewältigungsstrategien im Alltag wurden dabei spezifisch in Bezug auf die Auswirkungen auf ihre Lebenssituation, ihre Selbsteinschätzung als vulnerable Gruppe und ihre Ängste rund um COVID-19 sowie andere Sorgen untersucht.

In Bezug auf die Stimmungslage ging es den Befragten vor der Coronapandemie schon sehr gut, was sich über die Befragungszeitpunkte noch kontinuierlich verbesserte. Zwar gelten fehlende soziale Einbettung und Isolation als erhöhtes Risiko für psychische Belastungen insbesondere bei älteren Personen [22, 23]. Allerdings zeigten auch andere Studien, dass ältere Personen durch die Coronapandemie im Vergleich zu jüngeren Personen weniger psychisch belastet wurden [13, 22]. Die kaum beeinflussten Alltagsroutinen zu Hause und die breiten Unterstützungsangebote aus dem Umfeld mögen hier eine Rolle gespielt haben.

Die Resultate lassen erkennen, dass sich der Alltag der Befragten zu Hause durch die COVID-19-Schutzmaßnahmen wenig veränderte. Gewohnheiten und Aktivitäten, welche mehrheitlich bereits durch die Pensionierung etabliert wurden, konnten beibehalten werden. Die Beschäftigung mit Aktivitäten und Routinen wird in der Literatur als proaktive Bewältigungsstrategie genannt, um psychisch und physisch gesund zu bleiben [9], und wurde auch im Zusammenhang mit der Coronapandemie in anderen Studien bestätigt [11, 18]. Dass diese Routinen gleichsam schon Teil des Alltags waren, hat die Adaptation wahrscheinlich unterstützt.

Die Einschränkungen im sozialen Bereich und das Leiden deswegen waren deutlicher. Beschäftigungen außer Hause wurden zu Beginn auf Spaziergänge begrenzt, die Mehrheit blieb ansonsten daheim. Die Sotomo-Befragung [5] bestätigte, dass Personen über 65 deutlich weniger physisch nahe Kontakte hatten als der Rest der Schweizer Bevölkerung. In der vorliegenden Studie vermissten die Befragten die physischen Kontakte mit Familienmitgliedern und Freunden ausgesprochen. Die Kompensation über die sozialen Medien wurde als anstrengend und wenig zufriedenstellend empfunden.

Wegen der starken Empfehlung im ersten Lockdown der Coronapandemie, dass ältere Personen zu Hause bleiben sollten, boten nicht nur Familienmitglieder, sondern auch Nachbar*innen und verschiedene Gruppen Unterstützung an. Die Befragten schätzten diese Angebote, auch wenn teilweise die Einschränkung der eigenen Autonomie als schwierig erlebt wurde. Sie berichteten von Schuldgefühlen und vermissten Partizipationsmöglichkeiten. Die Aufopferung Jüngerer für Ältere kann bedeuten, dass sich Grenzen und Differenzen zwischen den Generationen verschärfen und v. a. auch bei älteren Personen Schuldgefühle und ein reduziertes Selbstwertgefühl die Folgen sind [12].

Ausdrücklich kritisiert wurde von den Befragten, dass bei der Definition der besonders gefährdeten Gruppe rigide und undifferenziert das Rentenalter und weniger der (schlechte) Gesundheitszustand einbezogen wurde – Kritik, die auch andere Studien bestätigen [19, 22]. Die Befragten machten sich Sorge um die Qualität der intergenerationellen Beziehungen, hatten sie doch aus ihrem Umfeld oder aus den Medien Berichte mitbekommen, in denen ältere Personen offen angefeindet wurden. Auch in anderen Studien wurden Bedenken um negative gesellschaftliche und wirtschaftliche Veränderungen im Ansehen von Älteren bei jungen Personen diskutiert [10]. Tatsächlich können simple Vereinheitlichungen und undifferenzierte Debatten in Bezug auf das Alter negative Stereotype aufrechterhalten und Altersdiskriminierung fördern [6, 20, 21, 23]. Geht man von einem multidimensionalen Alter und pluralistischen Lebensläufen aus [24] und beachtet, dass ältere Personen in Bezug auf Lebenserfahrungen, Kultur, genetische Prädisposition und Gesundheit keine homogene Gruppe sind [2], ist zu Beginn der Pandemie wahrscheinlich ein Teil der Personen über 65 Jahre zu rigoros aus dem öffentlichen Leben ausgeschlossen worden.

Zum erhöhten Erkrankungs- und Mortalitätsrisiko von älteren Personen äußerten sich die Befragten gelassen. Sie gingen zwar davon aus, dass eine Ansteckung mit dem Coronavirus für sie gefährlich sein könnte, gleichzeitig ersetzten sie das Wort „Angst“ mit dem Wort „Respekt“: Sie hätten nicht Angst vor einem schweren Verlauf, aber Respekt davor. Vielleicht haben diverse Lebenskrisen, von denen die meisten zu erzählen wissen, zu dieser Gelassenheit geführt. Außerdem verwiesen einige auf ihre Patientenverfügung und die Vorbereitung sowie Auseinandersetzung mit dem eigenen Tod.

Insgesamt scheinen die betroffenen Personen die Pandemie deutlich besser bewältigt zu haben, als der öffentliche Diskurs nahelegte. Ältere Personen wurden von einer gebrechlichen und hilflosen Seite dargestellt, bei der negative Stereotype des Alters, wie Verlust und Zerfall, in den Fokus gerieten. Dies ist problematisch, weil ein Großteil der älteren Personen bei guter Gesundheit ist und wertvolle Beiträge in der Gesellschaft leistet [8]. Diese Ergebnisse unterstreichen, dass es wichtig ist, die z. T. in der Öffentlichkeit und ebenfalls in der Mainstream-Gerontologie herrschenden negativen Annahmen über das Alter mit dem positiv gesinnten Blick der Kritischen Gerontologie zu konfrontieren und damit hoffentlich auch zur Veränderung der Einstellung beizutragen [1].

Zu den Limitationen der Studie gehört der kurze Zeitraum der Befragung. Außerdem konnten wir keine Bewohner von Alters- und Pflegeheimen interviewen, da der Zugang zu Altersheimen aufgrund der Coronapandemie sehr erschwert war. Nicht erhoben wurde der sozioökonomische Status, weshalb wir keine Aussagen in Bezug auf diese Verteilung machen können. Anvisiert wurde, eine anteilsmäßig gleiche Verteilung in Bezug auf die Wohnsituation, Geschlecht und Wohnort zu erhalten (Tab. 1). Die Wohnsituation (allein oder mit Partner*in lebend) war relevant in Bezug auf das Thema soziale Isolation, der Wohnort (Stadt, Land) in Bezug auf die Ansteckungsgefahr und die Möglichkeiten, nach draußen zu gehen.

## Fazit für die Praxis

- Ältere Personen verfügen über vielfältige Strategien, wie sie in einer Krise den Alltag bewältigen.
- Soziale Unterstützung von älteren Personen wird geschätzt, sollte aber immer auch deren Autonomiebedürfnisse antizipieren.
- Online-Kontakte werden als anstrengend empfunden und können die Face-to-Face-Kommunikation nicht befriedigend ersetzen.
- Die homogene Einteilung älterer Personen als Risikogruppe vernachlässigt deren Ressourcen und ist somit altersdiskriminierend.

## Supplementary Information




