# Supplementary material for: Specially protected or stereotyped? How persons older than 65 years experienced the COVID-19 pandemic
Source: Z Gerontol Geriatr. 2022 Sep 28;56(4):294–300. [Article in German] doi: 10.1007/s00391-022-02112-9 (PMC9517977; doi:10.1007/s00391-022-02112-9)
Supplement: Supplementary file 1 [file 391_2022_2112_MOESM1_ESM.docx]

**Besonders geschützt oder ausgestossen? Wie Personen über 65 Jahren die Corona-Pandemie erlebten**

**Fragebogen**

**Stimmungslage**

1. Erzählen Sie mal, wie es Ihnen zurzeit geht.

- -2: sehr schlecht
- -1: schlecht
- 0: geht so
- 1: gut
- 2: sehr gut

1. Wie ging es Ihnen vor Beginn der Corona-Krise? (auf einer Skala von -2 bis 2 zu beantworten) (nur t1):

- -2: sehr schlecht
- -1: schlecht
- 0: geht so
- 1: gut
- 2: sehr gut

**Körperliche Gesundheit**

1. Können Sie mir darüber berichten, wie ist Ihre körperliche Gesundheit? (von uns)
2. Jetzt weiss ich gar noch nicht, waren Sie allenfalls schon mit dem Coronavirus infiziert?

- Typische Symptome der COVID-Erkrankung
- Krankheitssymptome wie bei einer Erkältung oder Grippe
- Bisher keine Erkrankung und keine Symptome

1. Wie schätzen Sie die Gefährlichkeit des Corona-Virus (COVID-19) für sich selber ein?

- Ich mache mir keine Gedanken darüber
- Ich gehe von einem milden Verlauf aus
- Ich befürchte einen schweren Verlauf
- Ich befürchte einen tödlichen Ausgang

**Mobilität und Kontakte**

1. Wofür haben Sie diese Woche Ihre Wohnung/Ihr Haus verlassen?

- Einkaufen
- Spaziergänge
- Arbeit
- Sport
- Arztbesuch oder ähnliches
- Wanderungen
- Freunde/Bekannte in anderen Wohnungen treffen
- Freunde/Bekannte draussen treffen
- Ausflüge an andere Orte
- Ich bleibe zuhause

1. Welche Verkehrsmittel haben Sie vor der Corona-Krise (Anfang 2020) mehrmals pro Woche genutzt? (nur t1)

- Auto/Motorrad
- Zu Fuss
- Velo, E-Bike, Scooter
- Tram/Bus, Zug
- andere (Flugzeug, Taxi) (von uns)
- keine

1. Mit wie vielen Menschen, die nicht mit Ihnen zusammenleben, hatten Sie diese Woche näheren Kontakt (länger als 15 Minuten, näher als 2 Meter)? Denken Sie dabei (auch) an alle Begegnungen in der Nachbarschaft oder unterwegs.
   Anzahl Begegnungen:

- 0
- 1-2
- 3-5
- 6-10
- 11-20
- 21+

**Politik und Öffentlichkeit**

1. Wie schätzen sie die Massnahmen, die die persönliche Bewegungsfreiheit einschränken (Verbot von Ansammlungen mit mehr als fünf Personen, Besuchsverbot usw.) ein?

- Gehen viel zu wenig weit
- Gehen eher viel zu wenig weit
- Sind angemessen
- Gehen eher zu weit
- Gehen viel zu weit

1. Ganz grundsätzlich: Wie beurteilen Sie die Reaktionsgeschwindigkeit des Bundesrats in der Corona-Krise?

- Zu langsam
- Genau richtig
- Zu überhastet

1. Wie gross ist Ihr Vertrauen in die politische Führung der Schweiz in Bezug auf die Bewältigung der Corona-Krise?

- Sehr klein
- gering
- mittel
- gross
- Sehr gross

1. Wie nehmen Sie den zwischenmenschlichen Umgang in der aktuellen Situation wahr?

- Solidarisch
- Freundlich
- Egoistisch
- Misstrauen
- Interessiert
- Vertrauen
- Desinteressiert
- Aggressiv

**Krise und ihre Folgen**

1. Wie optimistisch oder pessimistisch sind Sie in Bezug auf den Verlauf und den Ausgang der Corona Krise?

- sehr pessimistisch
- pessimistisch
- neutral
- optimistisch
- sehr optimistisch

**t1: Thema Alltagsgestaltung und Alltagsbewältigung**

1. Erzählen Sie mir von Ihrem Alltag in ihrer Wohnung / im Altersheim. Ich möchte mir gerne vorstellen, wie ein üblicher Tag jetzt seit den Einschränkungen wegen der Corona-Krise bei Ihnen aussieht.
2. Was ist der Unterschied zum Alltag vor der Coronakrise?
3. Was fehlt Ihnen im Alltag? (*warten).* Was ist am schwierigsten zu ertragen?
4. Von wem und wofür haben Sie Hilfe oder Unterstützung erhalten? (Bsp. Familie, Gemeinde etc.)
5. Was fehlt Ihnen in Bezug auf Unterstützung? (Bsp. Einkaufen, Hilfe mit Smartphone Installation, um mit Enkel per Video telefonieren zu können u.v.m.)
6. Welche Unterstützung würden Sie sich wünschen? (*falls nicht erwähnt: Wie könnte diese Unterstützung Ihrer Meinung nach umgesetzt werden?*)
7. Was fällt Ihnen einfach, was ist schwierig? (*auf Antwort warten).*Haben Sie eine Strategie gefunden, mit dieser Situation umzugehen?
8. Wenn Sie mit jemandem zusammenwohnen: Hat sich das Zusammenleben verändert? (Evtl.: Haben Sie mehr Konflikte? (Thema Stress…)
9. Wir sind jetzt am Schluss von diesem Gespräch. Gibt es noch etwas, das ich noch nicht angesprochen haben, was Sie noch sagen möchten?

**t2: Hochrisikogruppe – was löst das aus?**

1. Wie erleben Sie die Zuweisung in die Hochrisikogruppe? (Denken Sie, dass Sie nicht dazu gehören?)
2. Es interessiert mich, wie Sie sich durch die besonderen Bestimmungen fühlen:

..eingeschränkt?

..geschützt?

..diskriminiert?

1. Welche Reaktionen der Bevölkerung erleben Sie? (*wenn nichts kommt:* negative Reaktionen, positive Reaktionen?)

Wenn nicht aus dem Haus geht: Nachbarschaft, Medien?

1. (*wenn nicht schon in 3. erwähnt)* Oder auf der anderen Seite: Haben Sie in dieser Zeit Beschuldigungen oder Vorurteil gegenüber älteren Personen wahrgenommen?
   *wenn nicht so viel erzählt hat: «Können Sie mir dazu noch etwas mehr erzählen? (Info: was, von wem, was Sie darüber denkt)*
2. Wie reagieren Sie auf diese Reaktionen?
3. (*wenn nicht schon in 3. erwähnt)* Kümmern sich Personen deshalb besonders um Sie?
4. Haben Sie persönlich Angst? *(Wenn jemand die Frage nicht zu verstehen scheint: Angst vor einem schweren Verlauf, heftigen Symptomen.)*
5. Was macht Ihnen besonders Angst? Wie gehen Sie mit Ihrer Angst um?
6. Ich habe kürzlich eine Aussage von einer Risikopatientin (65+) in der Zeitung gelesen: «Ich möchte lieber Enkel und Freude sehen, als ein paar Monate länger leben.»

Was denken Sie von dieser Aussage?

1. Wir sind jetzt am Schluss von diesem Gespräch. Gibt es noch etwas, das ich noch nicht angesprochen haben, was Sie noch sagen möchten?

**t3: Einschätzung der jetzigen Auswirkung**

1. Was denken Sie, verändert die jetzige Corona-Pandemie unsere Welt und wie verändert sie unsere Welt im sozialen Bereich? *Zuerst erzählen lassen.*

*Dann in weiteren Fragen die Punkte aufnehmen, die noch nicht erwähnt wurden. Folgende Punkte sollten vorkommen:*

- *Familien*
- *Kinder*
- *ältere Menschen*
- *Nachbarn*
- *Freunde*

1. Werden die Leute längerfristig anders miteinander umgehen? *(Solidarität / Egoismus?)*
2. Was denken Sie, wie verändert die jetzige Corona-Pandemie unsere Welt in Bezug auf die Politik und Wirtschaft in der Schweiz?

In Europa / ganze Welt?

1. Hat sich die ganze Corona-Krise darauf ausgewirkt, wie Sie über die Politik denken?
2. Befürchten Sie aufgrund der beschlossenen Massnahmen und der Geschwindigkeit der Lockerungen eine 2. Welle?
3. Gibt es etwas, was Sie besonders beschäftigt (falls nicht Thema Politik, Wirtschaft etc. erwähnt wird, kurz darauf aufmerksam machen). (Auch in Bezug auf die Zukunft)
4. Wir sind jetzt am Schluss von diesem Gespräch. Gibt es noch etwas, das ich noch nicht angesprochen haben, was Sie noch sagen möchten?

**t4: Erfahrungen von früheren Krisensituationen**

1. Sie haben möglicherweise schon andere Krisensituationen erlebt, global/national gesehen. Wenn Sie zurückdenken, welche Krisen kommen Ihnen in den Sinn?
2. Wie ging man früher mit Krisensituationen um (persönlich / gesellschaftlich / politisch)?
3. Gibt es einen Unterschied zu heute im Umgang mit Krisensituationen? *(Falls nicht angesprochen: Haben sich die Menschen anders geholfen?)*
4. Haben Sie das Gefühl, hilft Ihnen diese vergangene Situation (oder Situationen) für die Bewältigung der jetzigen Situation?
5. Welchen Rat würden Sie jüngeren Leuten zur Bewältigung der Situation mitgeben?
6. Wir sind jetzt am Schluss von diesem Gespräch. Gibt es noch etwas, das ich noch nicht angesprochen haben, was Sie noch sagen möchten?
